# Preparation 606

**Published:** 1910-08-20

**Authors:** 


### "PREPARATION 606."

The enterprise of the modern newspaper editor in catering for a sensation-loving public has long ago exploited the various fields of medicine and surgery in which the laity can be induced to take an interest. There is very good reason, indeed, for the instruction of everyone in the simple rules of hygiene; in correct notions with regard to cleanliness, fresh air, infant feeding, purity of food supply, temperance, and the avoidance of infections, especially that of tuberculosis. Popular medical science has much yet to accomplish in carrying out these and similar enterprises before it can have any justification for divagations into complicated medical researches. For some years, however, the pushful halfpenny press has from time to time kept its readers supplied with snippets of information as to the latest new and infallible cure, be it for cancer, consumption, or what not. In most cases these paragraphs describe the wonderful results obtained by some foreign medical man in the treatment of one or other of the fairly common diseases for which hitherto a certain cure is still to seek. Such paragraphs are open to many serious objections. First of all, they nearly all contain very gross overstatements, and in nine cases out of ten the supposed discovery soon turns out to be absolutely worthless. Next, even if there is a basis of accuracy underlying them, it is always probable that the truth will be mangled out of recognition when it must run the gauntlet of translator, reporter, and editorial expurgation. Again, even should the paragraph be irreproachable in accuracy (this generally requires that it should be more or less technical), experience proves that far more harm than good is generally caused. It is a commonplace event for practitioners attending a patient to receive either from friends, relatives, or the patient himself a newspaper clipping with a request for information about some alleged new treatment for the disease from which the patient is suffering. In any case the publication of medical research should be always in full; that is to say, in the columns of some recognised medical paper, since the general press cannot possibly afford the necessary space. Snippets and garbled extracts are of no value whatever where new medical discoveries are concerned, and have proved to be the source of perpetual worry and disappointment.

It is, therefore, with considerable distrust that the medical profession will naturally receive the telegrams recently printed about "Preparation 606." Even the *Times*, of which better things are expected, unfortunately seems to be imitating its younger but less reputable rivals in matters medical; for two paragraphs, from Berlin and Frankfort respectively, are published about this wonderful new arsenical compound which is alleged to have rescued already some two thousand sufferers from the grave. From other sentences in this report it would seem that syphilitic and parasyphilitic diseases are those for which the drug has principally been tried, and apparently it is Dr. Paul Ehrlich who has introduced it. With the complete collapse of the artificial boom in organic arsenic medication which spread over this country so recently from abroad, an attitude of scientific conservatism is essential, notwithstanding the great name by which "Preparation 606" is sponsored. But meanwhile the public will believe that, since the paragraph is in the *Times*, it must all be true, and there is a very fair chance that the profession will in consequence be forced into extensive use of a new German preparation which is only too likely to be as useless—though it is to be hoped not so harmful—as many of those which have preceded it.

